# Compact and low-power wireless headstage for electrocorticography recording of freely moving primates in a home cage

**DOI:** 10.3389/fnins.2025.1491844

**Published:** 2025-02-27

**Authors:** Taro Kaiju, Masato Inoue, Masayuki Hirata, Takafumi Suzuki

**Affiliations:** ^1^Center for Information and Neural Networks (CiNet), National Institute of Information and Communications Technology, and Osaka University, Suita, Japan; ^2^Department of Neurological Diagnosis and Restoration, Osaka University Graduate School of Medicine, Suita, Japan

**Keywords:** electrocorticography, electrophysiology, nonhuman primates, wireless recording, brain-machine (computer) interface

## Abstract

**Objective:**

Wireless electrocorticography (ECoG) recording from unrestrained nonhuman primates during behavioral tasks is a potent method for investigating higher-order brain functions over extended periods. However, conventional wireless neural recording devices have not been optimized for ECoG recording, and few devices have been tested on freely moving primates engaged in behavioral tasks within their home cages.

**Methods:**

We developed a compact, low-power, 32-channel wireless ECoG headstage specifically designed for neuroscience research. To evaluate its efficacy, we established a behavioral task setup within a home cage environment.

**Results:**

The developed headstage weighed merely 1.8 g and had compact dimensions of 25 mm × 16 mm × 4 mm. It was efficiently powered by a 100-mAh battery (weighing 3 g), enabling continuous recording for 8.5 h. The device successfully recorded data from an unrestrained monkey performing a center-out joystick task within its home cage.

**Conclusion:**

The device demonstrated excellent capability for recording ECoG data from freely moving primates in a home cage environment. This versatile device enhances task design freedom, decrease researchers’ workload, and enhances data collection efficiency.

## 1 Introduction

Electrophysiological recordings conducted during behavioral tasks in nonhuman primates (NHPs) are widely recognized as essential tools for elucidating brain function. NHPs exhibit substantial physical, behavioral, and cognitive similarities to humans, making them valuable models for understanding human brain function (Phillips et al., 2014). However, conventional tethered (wired) recording techniques possess numerous limitations. Unlike small animals such as rodents, NHPs are often restrained in primate chairs during electrophysiological recordings (McMillan et al., 2017). This restriction limits the animals’ ability to exhibit natural behaviors, necessitates considerable attention to their comfort, and reduces the duration of recording sessions. Additionally, tethered recordings carry the risk of noise contamination through cables and potential damage to the equipment caused by animals’ behavior (Won et al., 2023).

**FIGURE 1:**
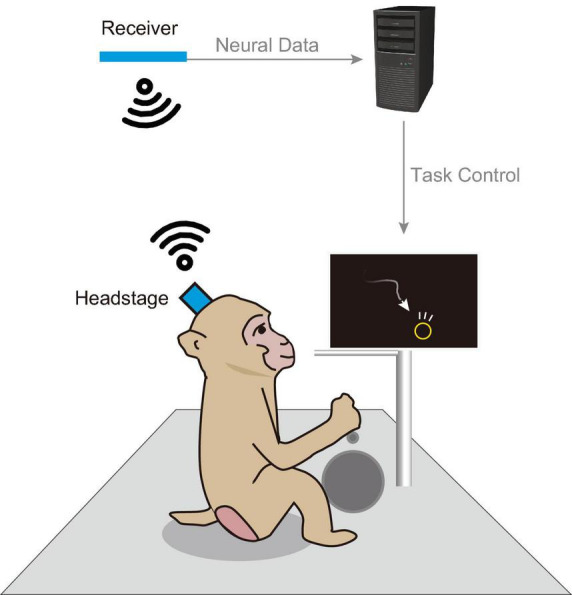
Schematic of an in-cage behavioral task. Schematic illustration of a behavioral task and wireless recording setup within a home cage. Visual stimuli were displayed on a monitor installed within the cage, and the animal engaged in behavioral tasks using a manipulandum (e.g., button, lever). Successful completion of a trial resulted in a liquid reward dispensed from a tube. During the task, a wireless headstage equipped with a neural electrode array recorded brain activity. Data captured by the headstage were transmitted to a nearby receiver and subsequently sent to a host PC for storage.

Therefore, there is a growing need for electrophysiological recordings under nonrestraint conditions. Figure 1 schematically illustrates wireless recording of a monkey engaged in a behavioral task. Wireless recording techniques allow animals to behave freely, thereby enhancing data collection efficiency. The increased freedom in task design facilitates the emergence of new scientific insights. This study aimed to develop a general-purpose wireless recording device in neuroscience research, with a particular focus on electrocorticography (ECoG). ECoG is a surface-based recording technique that avoids the need for cortical penetration and provides long-term signal stability and excellent spatiotemporal resolution (Belloir et al., 2023; Kaiju et al., 2021; Moon et al., 2024).

Several previous studies on wireless recording devices for large NHPs have successfully demonstrated recordings under unrestrained, freely moving conditions. However, most of these devices have not been optimized for ECoG recording. For example, devices optimized for specific electrodes, such as the Utah electrode (Fernandez-Leon et al., 2015; Foster et al., 2014; Yin et al., 2014) or microdrive (Zhou et al., 2019), have specialized connectors and headstage shapes, making it challenging to integrate them with user-preferred ECoG electrodes. Commercially available general-purpose wireless headstages (Berger et al., 2020) and custom high-channel-count devices (Schwarz et al., 2014) are not dependent on a specific electrode arrays. However, they exhibit greater power consumption compared to ECoG-specific devices and require large housings to protect the device during unrestrained use. Fully implantable devices (Borton et al., 2013; Romanelli et al., 2018) cannot accommodate user-preferred electrodes due to the integration of the electrode and body, necessitating a large, constantly powered cage or long charging times for the organism. Many other studies, including those focused on devices for rodents, have been published; however, continued validation is required to determine the suitability of these devices for use in large NHPs under freely moving conditions. In summary, existing devices face limitations related to electrode connectivity, power consumption, device size, and lack of validation in large animals.

To address this gap, the present study aimed to develop a wireless recording device optimized for ECoG neuroscience and demonstrate its efficacy in recording brain activity during unrestrained, cage-based behavioral tasks (Butler and Kennerley, 2019; Calapai et al., 2017; Griggs et al., 2021). The device maximizes battery life through its specialization in ECoG measurements and includes remote power management capabilities to ensure experimenter safety. Moreover, it offers flexible protection for electronics suitable for in-cage use. Its standard Omnetics connector and upright headstage design guarantee compatibility with various research electrodes, thereby enhancing its utility as a versatile tool for neuroscience research.

The first half of this paper provides a detailed description of the system and presents the results of a bench-top evaluation. The second half presents findings from a demonstration study involving monkeys. The device demonstrated its usefulness in conducting continuous and intermittent measurements over long periods, as well as in measuring brain activity during a visuomotor task.

This study marks a remarkable advancement in overcoming technical challenges associated with electrophysiological recordings during behavioral tasks, particularly in large animals such as macaques. It enables the observation of brain activity in more natural behavioral states, thereby alleviating the data collection burden on researchers. This contributes to a deeper understanding of brain function and holds promise for advancements in medicine and engineering.

## 2 Materials and methods

### 2.1 Device design and fabrication

#### 2.1.1 Headstage and receiver

**FIGURE 2:**
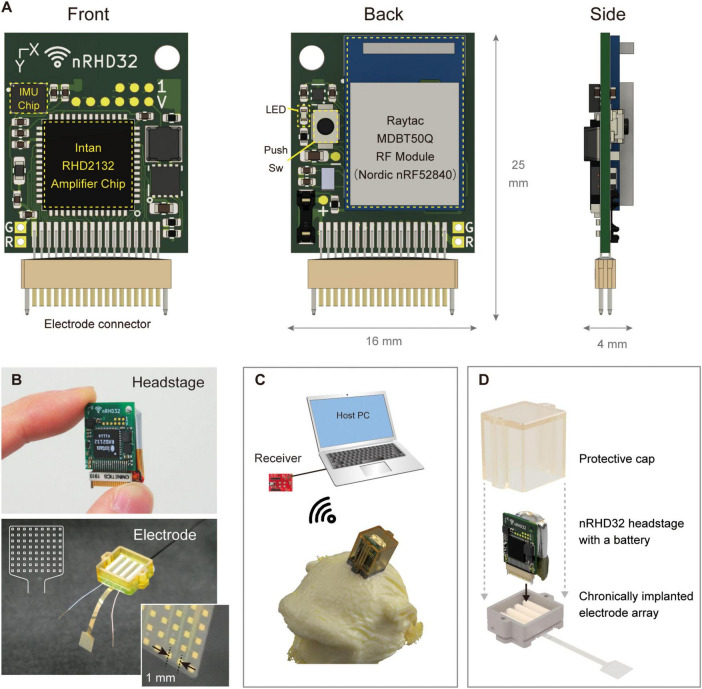
Device overview. **(A)** Pictures of the headstage: (left) front view showing the amplifier chip mounting side of the board, (center) back view depicting the RF module mounting side, and (right) side view showing additional details. The board top featured a universal hole for fixation. **(B)** Photographs of the (top) headstage and (bottom) electrode array. A schematic illustration of the electrode array is shown in the upper left corner of the photograph. **(C)** The receiver, host computer, and 3D illustration of the device mounted on the nonhuman primate head with a protective cap. **(D)** Assembly of the headstage, electrode array, and protective cap designed for use by nonhuman primates. The headstage and a lithium polymer battery (100 mAh) were installed on the chamber and covered with a protective cap. The flexible electrode array was electrically bonded to the PCB positioned on the bottom side of the chamber. The headstage was connected to only one of four connectors, enabling recording from 32 channels.

The developed wireless headstage comprised three core components: a 2.4 GHz band wireless module, a neural signal recording integrated circuit (IC) with 32 channels, and an inertial measurement unit (IMU) for acceleration and gyro measurement (Figures 2A, B). The wireless module (MDBT50Q-1MV2, Raytac Corporation, Taiwan) included a radio frequency system-on-chip (RFSoC) (nRF52840, Nordic Semiconductor, Trondheim, Norway) based on a Cortex-M4 microcontroller (MCU) (Arm Ltd., Cambridge, United Kingdom) with an operating clock of 64 MHz and internal RAM of 256 kB. This RFSoC supported various wireless protocols, including the Enhanced ShockBurst protocol proprietary to Nordic Semiconductor, chosen for its simple architecture and superior latency. The Enhanced ShockBurst protocol offered a data rate of 2 Mbps. The neural signal recording IC (RHD2132, Intan Technologies, CA, USA) was directly connected to the MCU via an 8 MHz serial peripheral interface (SPI) in a single-ended configuration. The IMU (ICM-42605, InvenSense Inc., CA, USA) was connected to the MCU through a 4 MHz SPI. A 36-pin miniature connector (NSD-36-DD-GS; Omnetics Connector Corporation, MN, USA), widely used in electrophysiology devices, was selected as the connector to electrode arrays. Power was supplied by a 100 mAh lithium-ion polymer (Li-Po) battery, delivering an output of 2.8–4.2 V. This voltage was regulated to a stable 3.7 V using a step-up/step-down DC-DC converter (TPS639000DSKR; Texas Instruments, TX, USA) and supplied to the main board. Additionally, a low-dropout regulator (TPS7A2033PDQNR; Texas Instruments, TX, USA) provided clean power at 3.3 V to the neural signal recording IC, ensuring optimal conditions for noise-sensitive neural recording. Firmware was developed using Nordic nRF5 SDK (Versions 17.0.2 and 17.1.0), and the MCU was programmed using J-Trace (SEGGER Microcontroller GmbH, Nordrhein-Westfalen, Germany). For data reception, an MDBT50Q-DB (Raytac Corporation, Taiwan), equipped with the same RF module as the headstage, was connected to the host PC via universal serial bus (USB) 2.0 (Figure 2C). Data transfer was performed using the Communications Device Class protocol. Power consumption was evaluated using the Nordic Power Profiler Kit 2 (Nordic Semiconductor, Trondheim, Norway).

#### 2.1.2 Electrode array

We developed a chronically implantable 72-channel ECoG electrode array for *in vivo* assessment of the developed headstage (Figure 2B). Parylene-C [poly(chloro-para-xylylene)] was used as the flexible substrate, with gold for electrode contacts and conductor paths. Each electrode contact measured 300 μm × 300 μm, spaced at 1,000 μm center-to-center, and arranged in eight rows and nine columns. The array had a thickness of 20 μm and was fabricated using a previously reported method (Kaiju et al., 2017). The fabricated array was connected to a PCB equipped with four Omnetics connectors (NPD-36-VV-GS; Omnetics Connector Corporation, MN, USA) via bump bonding. The entire assembly was then encapsulated in epoxy resin adhesive (Bond E Set; Konishi Co., Ltd., Osaka, Japan). Ground and reference electrodes were constructed from tip-exposed Teflon-coated silver wires with a conductor diameter of 0.254 mm (The Nilaco Corporation, Tokyo, Japan) or parylene-coated gold wires with a diameter of 0.15 mm. Details regarding the fabrication of custom protective chambers and caps (Figure 2D) are provided in the Supplementary methods.

### 2.2 Evaluation parameters

#### 2.2.1 Noise floor evaluation

For noise level evaluations, recordings were conducted for approximately 1 min with a configuration where the ground, reference, and all channels were shorted to the reference input of the headstage. Following the recording, the standard deviation of voltage fluctuations for each channel was calculated to determine the noise floor. The periodogram was computed using the pwelch() function in MATLAB, to estimate the average power spectral density over the 1-min period. A Hamming window with a segment length of 1,000 samples (1 s) and a 50% overlap between adjacent segments was applied. The fast Fourier transform (FFT) was performed with 1,024 points.

#### 2.2.2 Latency

For latency evaluation, an oscilloscope (DSO9204H, Agilent Technologies, CA, USA) was used to simultaneously probe the debug ports of both the headstage and receiver. The first trigger was generated at the headstage when the amplifier chip started sampling, and the second trigger was generated at the receiver upon completion of data queueing to the USB transfer buffer (Supplementary Figure 1). Probing was conducted for approximately 30 min with the host PC in a receiving state. Statistical analysis involved capturing 11,001 pairs of triggers using the delay measurement feature of the oscilloscope.

#### 2.2.3 Packet loss rate

The packet loss rate was assessed by positioning the headstage and receiver at specific distances from each other (0.5, 1.0, 2.0, 3.0, 4.0 m) within a cage room. For each distance, recordings were performed for 120 s (120,000 packets). The packet loss rate was determined by dividing the number of missing packets by the total number of transmitted packets. Missing packets were identified using time stamps added to the header of each packet by the headstage. This process was repeated three times at each distance to obtain the average packet loss rate.

### 2.3 Animal experiment

#### 2.3.1 Experimental conditions and animals

Three adult male macaque monkeys (*Macaca fuscata*, Monkey G: 7.4 kg, Monkey T: 12 kg, Monkey E: 10.1 kg) were employed to assess the developed device. All experimental protocols were conducted in accordance with the animal research guidelines of Osaka University Graduate School of Frontier Biosciences and Graduate School of Medicine, as well as the Guide for the Care and Use of Laboratory Animals issued by the National Institutes of Health, USA. This study was approved by the Animal Experiment Committee at Osaka University Graduate School of Frontier Biosciences (Approval number: FBS-23-007) and Graduate School of Medicine (Approval number: 05-059-001). Animals were sedated with ketamine (2.5 mg/kg) and medetomidine (0.1 mg/kg), intubated and maintained with isoflurane (1–4%, inhalation). Surgery was performed aseptically, with continuous monitoring of the animals’ conditions and vital signs. A craniotomy was performed around the primary somatosensory cortex (S1). A left craniotomy was conducted on Monkey G and Monkey E, while a bilateral craniotomy was performed on Monkey T. In Monkey T, the electrode implanted in the left hemisphere was evaluated in this study. After the dural opening, the electrode array was placed on the finger representation area, which was identified through intraoperative finger stimulation (cathodic square pulse: 2-mA amplitude, 0.2-ms pulse width, and 1,012-ms pulse interval) and mapping of somatosensory evoked potentials. Ground and reference electrodes were implanted in the space between the dura and the inner surface of the cranium. Carprofen (3 mg/kg, subcutaneous injection) was administered as an analgesic, and ceftriaxone sodium (25 mg/kg, intramuscular injection) was given as an antibiotic. Recordings were conducted using the 32 channels accessible from one of the four connectors.

#### 2.3.2 Comparison of wired and wireless recordings of spontaneous activity and somatosensory evoked potentials

Spontaneous activity and somatosensory evoked potentials (SEPs) were recorded in Monkey E under sedation with ketamine (2.5 mg/kg, intramuscular injection) and medetomidine (0.1 mg/kg, intramuscular injection). Recordings were performed first using the developed wireless device, followed by recordings with a commercial wired device (RHD 32-channel headstage and RHD2000 system, Intan Technologies, CA, USA). The sampling frequency was set to 1 kHz for both systems. For spontaneous activity recordings, the periodogram was calculated using the pwelch() function in MATLAB, estimating the average power spectral density (PSD) over a 170-s period. A Hamming window with a segment length of 1,000 samples (1 s) and a 50% overlap between adjacent segments was applied. The FFT was performed with 1,024 points.

For SEP recordings, coiled spring electrodes were placed around the index and little fingers of the right hand, with a counter electrode affixed to the right forearm. Conductive paste (Ten20, Weaver and Company, CO, USA) was applied between the electrodes and the skin to ensure proper contact. The electrodes were connected to an electrical stimulus generator (STG-4008; Multi Channel Systems, Germany), and cathodic square wave pulses (2-mA amplitude, 0.2-ms pulse width, and 1,012-ms pulse interval) were applied to the index and little fingers. The signals measured by each device were averaged over 50 trials in SEP recording. For each trial, the signal was extracted within a window from −40 to +40 ms, with time *t* = 0 ms corresponding to the stimulus onset. Baseline correction was performed by setting the average voltage from *t* = −40 to −1 ms to zero. Among the 32 recorded channels, the channel exhibiting the largest peak-to-peak difference between positive and negative peaks after stimulation was selected, and this difference was defined as the peak-to-peak value: P*_*evoked*_*. The signal-to-noise ratio (SNR) was calculated as SNR = P*_*evoked*_*/P*_*baseline*_*, where P*_*baseline*_* was the peak-to-peak value obtained from the pre-stimulus period.

#### 2.3.3 Continuous and intermittent recording test in the home cage

The wireless headstage was placed within a protective chamber, and recordings were conducted with the freely moving animal (Monkey G) in its home cage. The receiver antenna was positioned approximately 50 cm from the cage. During intermittent recording experiments, the device was kept in standby mode when not actively recording to reduce power consumption (details of this feature are described in Section “3.1 Specifications and benchmarks of the developed device” in the “3 Results” section). The beacon transmission interval, a user-adjustable parameter that determines power consumption during standby, was set to 3 min for this evaluation.

Time-frequency analysis was performed on the recorded data. Spectrograms were calculated using the spectrogram() function in MATLAB, estimating the power. A Hamming window with a segment length of 10,000 samples (10 s) and a 80% overlap between adjacent segments was applied. The FFT was performed with 4,096 points. For each frequency *f* and time *t*, the power P*_*f*,*t*_* was converted to a two-dimensional color map by calculating log_10_(P*_*f,t*_*).

#### 2.3.4 Center-out joystick task in the home cage

A visuomotor task, specifically a center-out joystick reaching task, was performed in Monkey T’s home cage. For visual stimuli, an 8-inch LCD monitor (LCD-8000VH3B, Century Corporation, Japan) and an analog output joystick (HF11R11, APEM Inc., France) were mounted on the cage’s sidewall. Additionally, a reward delivery tube was installed from the top side of the cage, extending downward. The receiver was positioned approximately 10 cm above the top of the cage.

During a task session, the pretrial waiting period concluded when the joystick stayed in the neutral position for 1,000 ms. Among eight radially arranged circles (targets, 16-mm diameter), positioned 24 mm from the center, one was randomly illuminated in green, indicating the desired direction for cursor movement. The animal was required to maintain the joystick in the neutral position for 1,000 ms after the target appeared. Subsequently, a square (6 mm × 6 mm) at the screen’s center changed from green to black, signaling the start of the movement phase (Go Cue). Following the Go Cue, the animal moved a circular cursor (4-mm diameter) across the screen using the joystick. A trial was deemed successful when the cursor contacted the target, at which point the monkey was rewarded with water. If the cursor did not reach the target within 60 s, the trial was considered unsuccessful, no reward was provided, and a new trial commenced.

Prior to data analysis, one channel exhibiting large amplitude fluctuations, suspected of damage, was excluded from the analysis. Common average reference processing was then applied. To isolate the high-gamma band signal, an 8th-order IIR band-pass filter spanning 80–250 Hz was used. Due to packet loss in the wirelessly recorded data, missing segments were replaced with zeros before filtering. The squared value of the signal was then calculated and smoothed using a moving average with a 100-ms window. Specifically, for time *t* = T, samples from *t* = T–99 to T were used for the moving average calculation.

Signals corresponding to each trial were extracted relative to the joystick movement onset (*t* = 0) for subsequent visualization. A detailed explanation of the trial selection process is provided in the Supplementary methods (“*Center-Out Joystick Task: Trials Selection*”).

### 2.4 Statistical analysis

Statistical tests were performed using Welch’s *t*-test or the Wilcoxon rank sum test. A significance threshold of 0.05 was applied for all tests, with two-tailed testing. Statistical analyses were performed in MATLAB R2020b. Unless otherwise specified, data are presented as mean ± standard deviation.

## 3 Results

### 3.1 Specifications and benchmarks of the developed device

**FIGURE 3:**
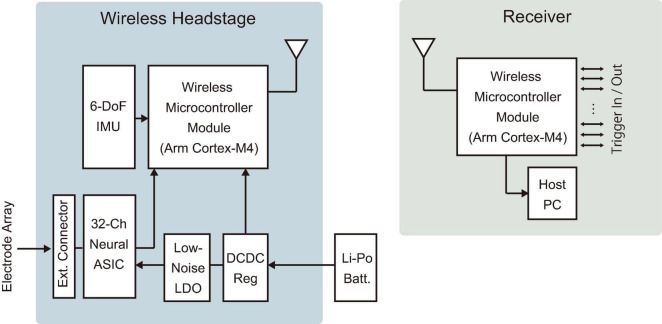
Device architecture. Diagrams of the wireless headstage and receiver. An electrode array was connected to the external connector of the headstage. A Li-Po battery was connected via an onboard connector on the headstage. IMU, inertial measurement unit; LDO, low-dropout regulator; DCDC Reg, DC-DC switching regulator; Batt., battery.

**FIGURE 4:**
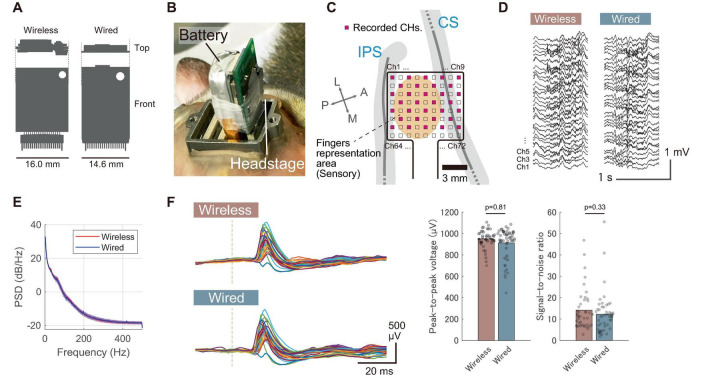
Comparison with a wired recording device. **(A)** Size comparison of the wireless headstage with a commercially available wired headstage (RHD 32-channel headstage, Intan Technologies). **(B)** Photograph showing the wireless headstage and battery installed on Monkey E’s head in the chamber, with the headstage connected to the first connector from the right. **(C)** Diagram displaying the anatomical placement of electrodes in the postcentral gyrus, with 32 out of 72 electrodes recorded (shown in pink). **(D)** Representative waveform of spontaneous activity recorded under sedation. **(E)** Power spectrum of spontaneous activity recorded under sedation with both wireless and wired devices. Solid lines represent the mean across 32 channels, and shaded areas indicate the standard deviation. **(F)** Somatosensory evoked potentials (SEPs) recorded from a sedated animal with both wireless and wired devices. The results were obtained from index finger stimulation. (Left) Representative unaveraged SEP waveforms obtained from a single stimulus. Different colored traces indicate signals recorded by the individual channels. Gray vertical dashed lines represent stimulus timing. (Right) Comparison of peak-to-peak voltages and SNRs between wireless and wired recordings obtained from 50 stimuli. Each dot represents the response from an individual stimulus, and bar plots indicate the mean. *P*-values were calculated using the Wilcoxon rank sum test. *P*-values are two-tailed.

The developed device features a compact and lightweight design, enabling simultaneous recording and transmission of 32 channels with a simple device architecture (Figure 3). Table 1 compares the specifications of the developed device with those of commercially available wireless headstages, while Supplementary Table 1 summarizes various validated wireless devices used with large, freely moving primates. The headstage measures 25 mm × 16 mm × 4 mm and weighs only 1.8 g (excluding the battery). Its compact size and lightweight design are comparable to the commercial wired headstage (Intan RHD 32-channel headstage), which measures 26 mm × 15 mm × 2.7 mm and weighs 1.0 g (Figure 4A).

**TABLE 1:** Device specifications and comparison with other commercial headstages.

	This work	W2100-HS32 (Multichannel Systems)	CerePlex W (Blackrock Microsystems)	W64 (Triangle BioSystems)
Electrode connector	Omnetics	Omnetics	CerePort	Omnetics
Channel count	32	32	96	63
Sample rate (kHz)	1	20	24.5	50
Size (mm^3^)	25.1 × 16.1 × 4.3^1^	15.5 × 15.5 × 6.5^2^	32.5 × 32.5 × 21^3^	25.0 × 20.5 × 14.2^4^
Weight	1.8 g^5^	3.6 g^6^	26.4 g^7^	5.0 g^8^
Resolution	16 bits	16 bits	12 bits	n/a^9^
Input referred noise	2.4 μVrms^10^	< 1.9 μVrms	< 3 μVrms	8.3 μVrms^11^
Battery life (hours)	8.5^12^	1.8^12^	3.5^13^	3.5–4.2^14^
Power (Run)	47 mW^12^	209 mW^12^	422 mW^13^,^15^	48 mW^14^
Power (Standby)	0.33 mW^16^	24.8 mW	–^17^	–^17^

^1^Without a battery and device housing.

^2^Without a battery and antenna.

^3^With a 400-mAh built-in battery and housing.

^4^With a 60-mAh built-in battery and dipped package.

^5^Without device housing and battery.

^6^Without a battery.

^7^With a 400-mAh built-in battery.

^8^With a 60-mAh built-in battery.

^9^W64 is an analog headstage.

^10^Conforms to RHD2132 amplifier chip datasheet.

^11^For 1 Hz- 7 kHz frequency

^12^Operated at 32 channels, 1 kSps with a 100-mAh battery.

^13^Operated with a 400-mAh built-in battery.

^14^Operated with a 60-mAh built-in battery.

^15^Estimated power consumption from battery life.

^16^Beacon interval was set to 30 s.

^17^Not provided.

The developed device demonstrated good power efficiency, with an average power consumption of 13 mA during wireless transmission at the maximum output power of 8 dBm (46 mW at 3.7 V operation, Supplementary Figure 2A). This is approximately one-fourth of the power consumption of a commercial device capable of recording up to spike bands when configured for ECoG-band recordings (Multichannel Systems W2100-HS32, 209 mW with 32 channels at 1 kHz sampling operation). To reduce the frequency of battery recharging or replacement for awake, large animals, the device was includes a low-power standby mode with an average power consumption of 0.09 mA (0.3 mW at 3.7 V operation, Supplementary Figure 2A). Effective battery management can be achieved by switching the device to standby mode when recording is unnecessary. This feature is particularly beneficial for standard neuroscience experimental designs, which typically involve 1–2 h recording sessions conducted daily over extended periods.

Given the challenges of accessing headstages attached to awake animals’ heads, particularly larger animals within their home cages, the developed device incorporates a feature that enables full remote control over its operational modes (run/standby). This functionality was implemented using polling, where the headstage intermittently transmits beacons at user-defined intervals. Recording and RF transmission begin upon detecting a receiver in receiving mode (Supplementary Figure 2B). This design eliminates the need for physical interaction with an animal, which is typically required with other wireless devices (e.g., pressing an ON/OFF switch, sending commands via infrared remote controllers, or operating a magnetic switch with a magnetic wand). The trade-off between the beacon interval and battery consumption is illustrated in Supplementary Figure 2C, showing the relationship between standby power consumption and the estimated standby duration at various beacon intervals. Additionally, the main board includes a push button that allows users to initiate recording immediately (Figure 2A).

The developed device demonstrated low latency and jitter, ensuring precise timing synchronization with external devices. The measured latency—from just before the amplifier sampling begins at the headstage to just after the data transfer is queued to the host PC at the receiver—was 788.1 ± 2.8 μs (mean ± standard deviation, 11,001 measurements). The maximum and minimum latencies recorded were 790.0 and 777.3 μs, respectively. These results confirm that the device exhibits sufficiently low latency and jitter for real-time applications such as closed-loop experiments and precise temporal synchronization. It is important to note that this latency measurement does not include the USB data transfer, data processing, or visualization in the host PC, as these latencies are highly dependent on the user’s application and environment (e.g., operating system).

The device also demonstrated a low packet loss rate, which is crucial for maintaining high-fidelity wireless communication. Supplementary Figure 2D illustrates the packet loss rate observed in the animal room. The packet loss rate remained consistently low across the tested distances ranging from 0.5 to 4.0 m, reflecting reliable performance for a freely moving animal in a cage setting.

The developed device achieved a low noise level comparable to that of wired devices. Supplementary Figure 3 illustrates the results of a noise floor evaluation that was conducted using a commercially available wired device (RHD 32-channel headstage) in a benchtop setup. The wireless headstage demonstrated a slightly higher noise floor compared to the wired headstage (Wireless: 2.7 ± 0.15 μV, Wired: 2.5 ± 0.18 μV, **p < 0.01, mean ± standard deviation across all 32 channels, Supplementary Figure 3D). However, these results confirm that the wireless device achieved a noise level nearly as low as that of the wired device. A significant factor contributing to the low noise floor was the stable power supply to the amplifier chip. The measured supply voltages inside the chip were 3.48 V and 3.31 V for the wired and wireless devices, respectively, with standard deviations of 370 μV and 510 μV over a 1-min period. These results indicate that the fluctuation of the internal voltage was sufficiently low to maintain a stable noise floor.

### 3.2 *In vivo* performance: comparison with a wired device

To evaluate *in vivo* performance, we compared the signal quality between the developed wireless and wired headstages. These headstages were alternately connected to an ECoG electrode array chronically implanted in the somatosensory cortex of a sedated animal (Monkey E). Figure 4B shows a photograph of the wireless headstage connected to the electrode connector, while Figure 4C displays the electrode location on the primary somatosensory cortex. Figure 4D illustrates the spontaneous brain activity recorded under sedation, showing spontaneous oscillatory activity and slow cortical potentials (Breshears et al., 2012) in both recording conditions. Figure 4E compares the resulting periodograms from each recording. The frequency characteristics were largely comparable between the two headstages.

To further characterize signal quality, we measured somatosensory evoked potentials (SEPs) and compared the results to those obtained with a wired device. As shown in Figure 4F, the wireless device successfully recorded SEPs elicited by a single stimulus, demonstrating comparable performance to the wired device. The peak-to-peak values of SEPs recorded with the wireless and wired devices were 949 ± 88 μV and 914 ± 149 μV, respectively, while the signal-to-noise ratios (SNRs) were 14.4 ± 9.8 and 12.4 ± 9.4, respectively (mean ± standard deviation, *n* = 50 responses). These results were obtained without averaging, indicating that the developed device has sufficient signal quality to capture single-shot cortical activation. Additionally, our electrode array featured hardware-level sub-sampling functionality achieved by interchanging the wiring between connectors. This capability enabled sampling across the entire array, despite the wireless device being limited to 32 wireless recording channels (Supplementary Figure 4A). We successfully captured the distribution of SEPs across the entire array, demonstrating performance comparable to the visualizing capabilities of a wired device with 72 channels (Supplementary Figure 4B).

### 3.3 *In vivo* performance: recording of a freely moving animal

**FIGURE 5:**
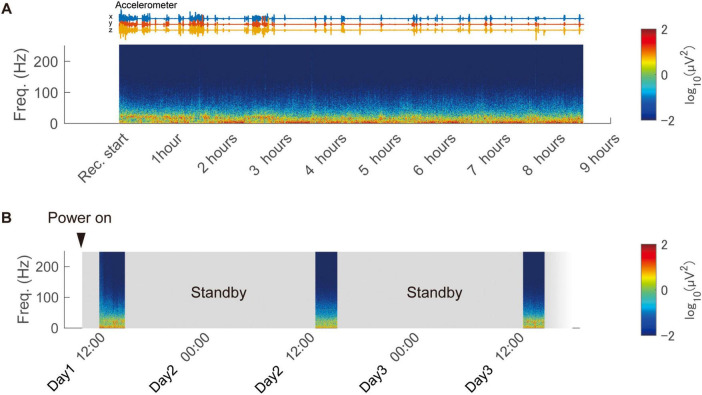
Continuous and intermittent in-cage recording using the wireless device. **(A)** Continuous recording from a freely moving animal in the home cage using the developed device. The spectrogram of the recorded signal is displayed, obtained from a representative channel. **(B)** Remote-controlled intermittent recording over three days using the low-power standby feature.

We assessed the long-duration recording performance of the wireless device in an awake and freely moving animal housed in a home cage (Monkey G). To evaluate continuous operation, we conducted recording using a fully charged 100 mAh battery, achieving 8.5 h of continuous recording (Figure 5A). Additionally, intermittent recording was tested by remotely switching the device to low-power standby mode when not in use. This setup enabled intermittent recording sessions of 2.5 h per day for three consecutive days, without requiring battery replacement or direct interaction with the animal (Figure 5B and Supplementary Figure 5). These results demonstrate the device’s low power consumption and flexibility in accommodating various recording schedules, including long-duration and intermittent sessions.

**FIGURE 6:**
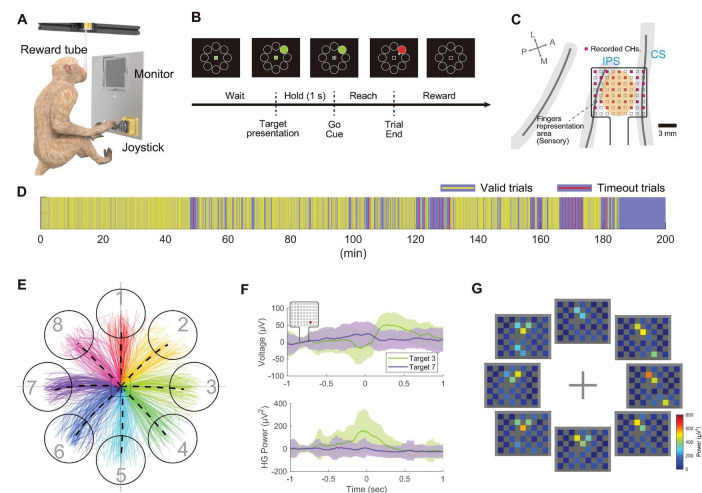
Wireless recording during the center-out joystick task in the cage. **(A)** Schematic of the behavioral task environment in the home cage. **(B)** Timeline of the center-out joystick task, illustrating the monitor display with the center mark, surrounding targets, and a cursor. **(C)** Diagram displaying the anatomical placement of electrodes in the postcentral gyrus. Out of 72 electrodes, 32 electrodes (shown in pink) were measured wirelessly. **(D)** Visual representation of trial progression during the recording session, where yellow bars indicate valid trials (target reached within the time limit), and red bars signify timed-out trials. **(E)** Diagram of cursor movement trajectories, with eight different colored lines representing trajectories to the eight targets. **(F)** Temporal changes in recorded potentials (top) and high-gamma band power (bottom) during movements toward Target 3 and Target 7, aligned to the moment the joystick movement was detected (*t* = 0). Solid lines represent the mean of all trials, with shading indicating the standard deviation (*n* = 94 trials). The representative channel is highlighted with a red square in the top left corner. **(G)** Diagram showing mean high-gamma band activity from –100 ms to +100 ms relative to joystick movement onset across all channels.

To validate the device’s capability to measure brain activity during behavioral tasks, an 8-direction center-out reaching task using a joystick was conducted (Figures 6A, B, Monkey T). Electrodes were placed in the digit representation area of the postcentral gyrus (Figure 6C). Over a behavioral session lasting 185 min, the monkey autonomously performed 1,589 trials at its own pace, taking breaks as needed (Figure 6D). Figure 6E illustrates the cursor trajectories for each target direction during the task. Analysis of broadband signals and high-gamma band power aligned with joystick movements is shown in Figure 6F. Modulations in broadband signals and high-gamma band power were observed around *t* = 0 (ms), with variations in magnitude depending on the target direction.

Figure 6G visualizes the distribution of high-gamma band power across the electrode array, indicating prominent power increases in channels over the postcentral gyrus, modulated by the target direction. To further interpret the response patches observed during the joystick movement task, digit-specific SEP response patches were overlaid on the high-gamma band activity map obtained during the motor task (Supplementary Figure 6). During the task, high-gamma band activity was predominantly observed in the D1 patch across all target directions. However, for certain target directions, such as Targets 3, 4, and 8, additional activity was also observed in the D4 and D5 patches.

In measurements from freely moving animals, packet loss can occur due to factors such as subject movement, multipath fading and electromagnetic interference, potentially affecting signal quality (Yin et al., 2014; Zheng et al., 2024). An analysis of packet loss across all 1,376 recorded trials revealed that 86.1% (1,185 trials) experienced no loss between the start of the inter-trial interval and the trial end, while only 6.4% (88 trials) experienced a single packet loss (each packet holds data for 1 ms) (Supplementary Figure 7). Additionally, the overall packet loss rate during the 151-min recording session was 0.07%, with the longest loss being 160 packets (160 ms). These results empirically demonstrate that reliable wireless communication can be maintained even during behavioral tasks in a nonrestrained environment within a cage.

## 4 Discussion

In this study, we successfully developed a new ECoG wireless device that is compact (25 mm × 16 mm × 4 mm), lightweight (1.8 g), operates for long durations (8.5 h with a 100 mAh battery), and has low latency (0.8 ms), facilitating long-term recording from freely moving NHPs. Moreover, we successfully recorded ECoG signals from a freely moving animal engaged in visuomotor tasks. To the best of our knowledge, this is one of the few successful demonstrations of ECoG recording in freely moving NHPs engaged in visuomotor tasks within their home cages. Previous wireless ECoG recordings from NHPs were typically constrained to animals seated in monkey chairs or head-fixed conditions, (Hu et al., 2018; Mollazadeh et al., 2011; Yan et al., 2023) or conducted under anesthesia (Ghomashchi et al., 2014). Such approaches left uncertainties regarding their applicability for recordings during unrestrained behavioral tasks.

The key features of this device include its low power consumption and compact, lightweight design. These features were achieved by restricting the recording bandwidth to below 500 Hz, optimized for ECoG/LFP recordings, and employing a simple architecture where a power-efficient, general-purpose RF microcontroller directly manages the neural recording chip. Consequently, compared to existing devices supporting spike band recordings (Foster et al., 2014; Liu et al., 2016; Schwarz et al., 2014; Yin et al., 2014), the size of the headstage and battery has been significantly reduced.

The lightweight and compact nature of this device is not an absolute requirement for successful recording from large NHPs like macaques. For example, previous studies have demonstrated the feasibility of using large-capacity batteries (5,200 mAh, ∼80 g) for long-term recording (Luan et al., 2018) or employing larger devices comparable in size to the diameter of the skull for high-channel count recording (Schwarz et al., 2014). However, we believe that reducing the device’s weight and size offers several notable advantages, particularly for in-cage recordings. A lighter device reduces physical stress on the animal, enhancing comfort and potentially improving compliance during behavioral tasks. Additionally, reduced weight allows for the use of larger-capacity batteries, enabling longer recording durations when necessary. The compact size further minimizes the risk of mechanical damage by reducing impact forces during collisions within the home cage. Therefore, the lightweight and compact form factor represents a significant advantage of this device.

The device was optimized for ECoG recording from larger NHPs during behavioral tasks within their home cage environment, yet its versatility extends beyond this specific application. It can be used for recording local field potentials (LFP, ∼300 Hz) from intracortical electrodes because the device supports 1-kHz sampling. Its compact, lightweight design renders it suitable for use with small animals, such as rodents. Furthermore, its high transmission power (+8 dBm) enhances its applicability across diverse environmental settings. Additionally, the adoption of readily replaceable batteries, instead of requiring external charging, and the use of standard Omnetics connectors have enhanced usability in neuroscience research.

The receiver setup for this device can be easily configured using a Windows-based laptop (or a single-board computer) and a single USB receiver board (Figure 2C), making it easy to use even in animal housing rooms where installing conventional and expensive neurophysiology systems may pose challenges. Despite its simple configuration, the packet loss rate during behavioral task measurements was only 0.07%, a better value compared to a previous study using four antennas for in-cage recordings (mean: 1.5%, best session: < 0.1%) (Hansmeyer et al., 2022). This result indicates the excellent performance of the device. However, this low loss rate may also be attributed to factors such as the device’s lower data rate, the antenna being positioned relatively close to the animal (∼30 cm), and the joystick task providing more stable head positioning compared to reaching tasks in prior studies.

Using this device, we successfully recorded high-density (32 channels/80 mm^2^) ECoG signals from the S1 region of a macaque performing a joystick-based behavioral task. As shown in Supplementary Figure 6D, high-gamma band activity during motor tasks partially overlapped with response patches for individual digits in the S1 region, with spatial patterns varying depending on the target direction. These direction-dependent results may reflect fine tactile or proprioceptive inputs during joystick manipulation. Additionally, it is known that S1 encodes not only afferent signals like tactile and proprioceptive information but also arm and hand kinematics (London and Miller, 2013). Such information may be reflected in high-gamma band activity measured from the cortical surface both before and during movement (Umeda et al., 2019). However, this study did not include simultaneous motor cortex recordings or motion capture analysis, limiting our ability to distinguish between sensory and motor contributions to S1 activity. Combining this headstage with advanced markerless motion capture systems (Mathis et al., 2018), which have seen significant progress in recent years, presents a promising approach to gaining deeper insights into the neural dynamics underlying motor tasks in unrestrained animals.

This study has other limitations. It was conducted concurrently with long-term evaluations of ECoG quality, necessitating electrode placement in the somatosensory cortex rather than in the motor cortex typically targeted in motor neuroscience. Additionally, despite the array having 72 channels, only 32 channels were measurable due to current device version limitations in RF bandwidth. However, visuomotor task measurements revealed distinct modulation of brain activity corresponding to cursor direction. We successfully captured overall activity patterns of the array using a limited number of channels through a strategically designed electrode array. These findings confirm the device’s functionality and its applicability to freely moving animals. In the future, we will focus on developing newer versions that support greater channel counts of the same size, leveraging advancements in neural ASIC and RF technology. This approach aims to enable more detailed analysis of complex brain activity patterns by researchers.

## 5 Conclusion

We developed a wireless ECoG recording device designed for large NHPs freely moving within their home cages. Our device successfully performed recordings from monkeys engaged in visuomotor tasks without restraint. Its high versatility makes it a valuable tool for neuroscience research, promising substantial contributions to demanding endeavors such as long-term closed-loop experiments, understanding the neural underpinnings of natural and high-degree-of-freedom body control, and enabling simultaneous recordings from multiple animals.

## Author contribuitons

TK: Conceptualization, Data curation, Formal Analysis, Funding acquisition, Investigation, Methodology, Project administration, Resources, Software, Validation, Visualization, Writing – original draft, Writing – review and editing. MI: Data curation, Investigation, Writing – review and editing. MH: Data curation, Investigation, Writing – review and editing, Funding acquisition, Supervision. TS: Conceptualization, Data curation, Funding acquisition, Investigation, Project administration, Supervision, Writing – review and editing.

## Data Availability

The raw data supporting the conclusions of this article will be made available by the authors, without undue reservation.
