# Supplementary material for: Compact and low-power wireless headstage for electrocorticography recording of freely moving primates in a home cage
Source: Front Neurosci. 2025 Feb 27;19:1491844. doi: 10.3389/fnins.2025.1491844 (PMC11903461; doi:10.3389/fnins.2025.1491844)
Supplement: Supplementary file 1 [file Data_Sheet_1.pdf]

Supplementary Methods

Device Design and Fabrication: Protective Chamber

A custom chamber was developed to protect the base of the electrode array, PCB with connectors, wireless headstage, and battery mounted on the animal's head (**Figures 2C and 2D**). The chamber consisted of a base and a cap. The base (22 mm × 20 mm × 9 mm) was made from machined polyetherimide plastic or 3D-printed stainless steel. The cap (22 mm × 20 mm × 24 mm) was produced using a 3D printer (AGILISTA-3200; Keyence, Osaka, Japan) with a urethane-based resin (AR-M2; Keyence, Osaka, Japan). The chamber was designed using 3D CAD software Fusion 360 (Autodesk Inc., CA, USA).

Center-Out Joystick Task : Trials selection

A total of 1,589 trials were performed in a behavioral session lasting 185 minutes. Neural recording was conducted for 151 minutes of the behavioral session, resulting in 1,376 trials with neural data. To focus on typical movements, 1,021 trials completed within 1,200 ms from the Go Cue to the target reach were selected. Among these, 901 trials without packet loss in the interval from −1.5 seconds to +1.5 seconds relative to movement onset were retained to ensure high-fidelity data analysis. To equalize trial counts across the eight targets, excess trials from each target were sequentially removed, matching the count of the target with the fewest valid trials (Target 4 with 94 trials). This exclusion was applied from the end of the recording session.

**A**: Behavioral session duration	185	min.	
**B**: Total number of trials in **A**	1,589	trials	(Incl. 19 timeout trials)
**C**: Neural recording duration in **A**	151	min.	
**D**: Number of trials with neural recording in **C**	1,376	trials	
**E**: Number of trials completed in <1200 ms among **D**	1,021	trials	
**F**: Number of trials without packet loss, during the interval from -1.5 seconds to +1.5 seconds relative to movement onset, among those in **E**.	901	trials	
**G**: Target direction with minimum trial counts among **F**	135	deg.	(Target 4)
**H**: Number of trial counts for **G** (Target 4)	94	trials	
**I**: Number of trials analyzed in **Figure 6E, 6F, and 6G**	752	trials	(94 trials × 8 directions)

Correspondence Between Visuomotor Task and Somatosensory Mapping

Somatosensory evoked potentials (SEPs) were recorded in Monkey T under sedation with ketamine (2.5 mg/kg, intramuscular injection) and medetomidine (0.1 mg/kg, intramuscular injection). Using a wired recording system, data collection followed the SEP recording protocol described in Methods Section 2.3.2. Electrodes were wrapped around all fingers, and stimuli (0.5, 1, and 2 mA) were randomly applied 50 times per finger and each intensity. Sampling was performed at 20 kHz. Clear SEPs were observed with 2 mA stimulation, and subsequent analysis focused on this data.

After resampling to 3 kHz, an 8th-order IIR band-pass filter spanning 80–250 Hz was applied to isolate the high-gamma band signal. Using the stimulus onset (t=0) as the reference, the mean squared signal value within the interval t=18±1.7 ms was calculated to determine evoked power for each channel. The evoked power was normalized by subtracting the mean and dividing by the standard deviation across all channels. The normalized evoked powers were rearranged into an 8×9 grid based on the electrode array, and contours corresponding to normalized response values of 1 were determined using MATLAB's contour() function. These contours were overlaid on high-gamma activity maps obtained during the task to create correspondence diagrams (**Supplementary Figure 7**).

Supplementary Figures

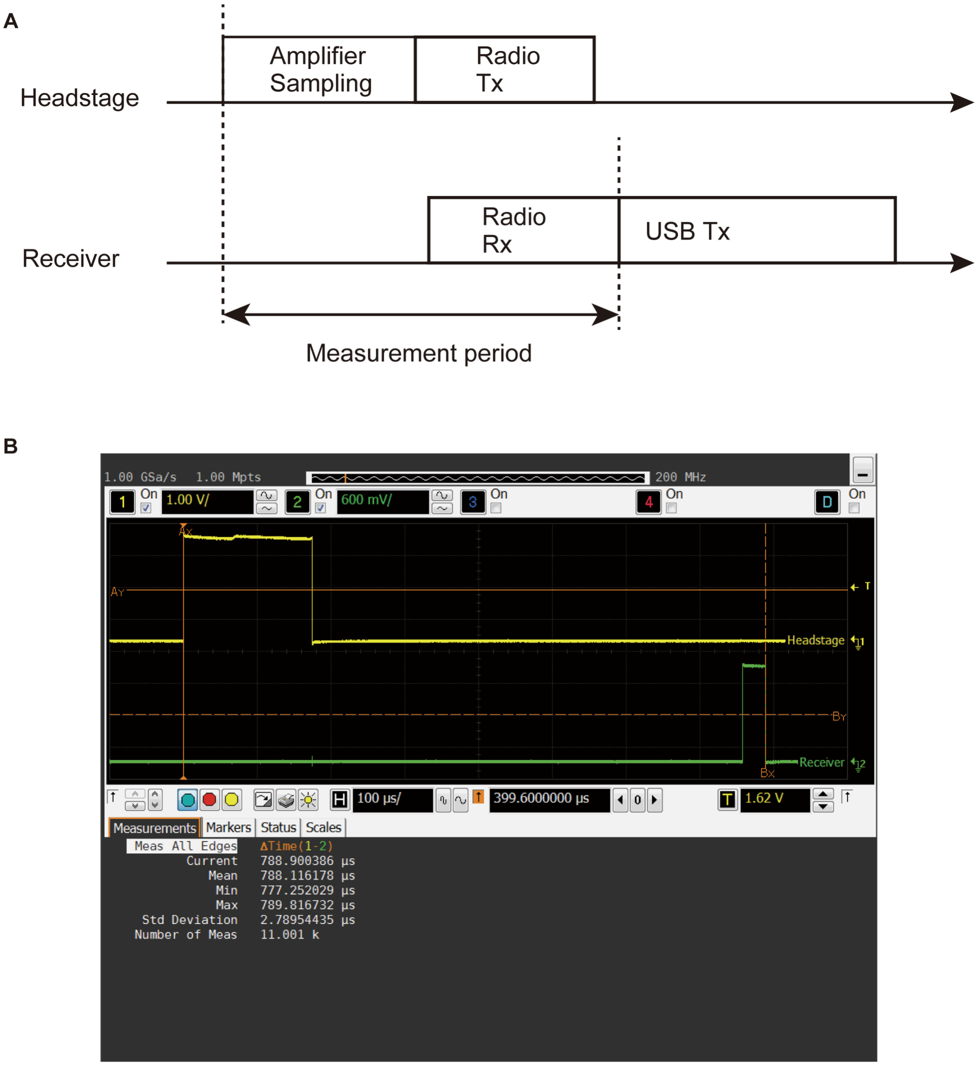

Supplementary Figure 1. Evaluation of the device latency.

A: Schematic of the evaluation. The device's latency was measured from just before the amplifier sampling to just after the USB data transfer queueing. **B**: Latency measurement results. The yellow trigger at the top of the screen was produced by the headstage, and the green trigger below was produced by the receiver. Statistical details are presented at the bottom of the screen.

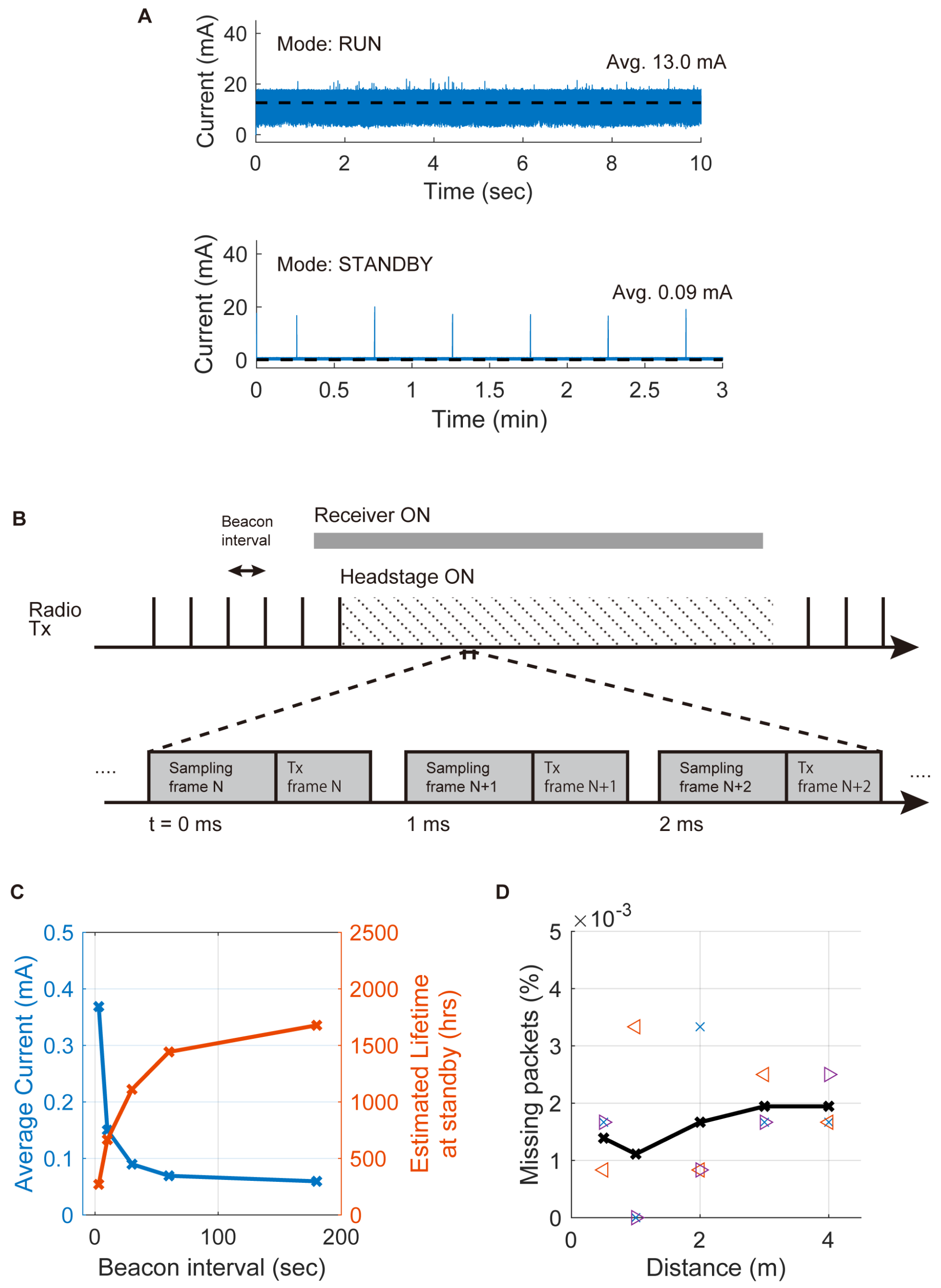

Supplementary Figure 2. Specifications of the developed device.

A: Measured current consumption during operation (top) and standby (bottom). The black dotted line represents the average power consumption. Pulse currents associated with beacon firing were observed at 30-s intervals in the standby state. **B**: (Top) Radio transmission schematic of the headstage in the standby and running states. Beacons were transmitted at user-defined intervals during standby. Upon receiver activation for recording, the headstage transitioned to the running state, transmitting data continuously at 1-ms intervals. The headstage returned to the standby state when the receiver was deactivated. (Bottom) Sampling cycle schematic of the headstage during the running state. Signal and subsequent transmissions were sampled in 1-ms cycles. **C**: Relationships between the standby beacon interval and average power consumption, as well as between the beacon interval and estimated battery lifetime in the standby state. The lifetime was estimated from average power consumption at 3.7 V with a 100-mAh battery capacity. **D**: Packet loss rate in the cage room. The horizontal axis shows the distance between the headstage and receiver, while the vertical axis represents the packet loss rate. The solid line represents the average packet loss rate observed at each distance. Differences in markers represent data from distinct trials.

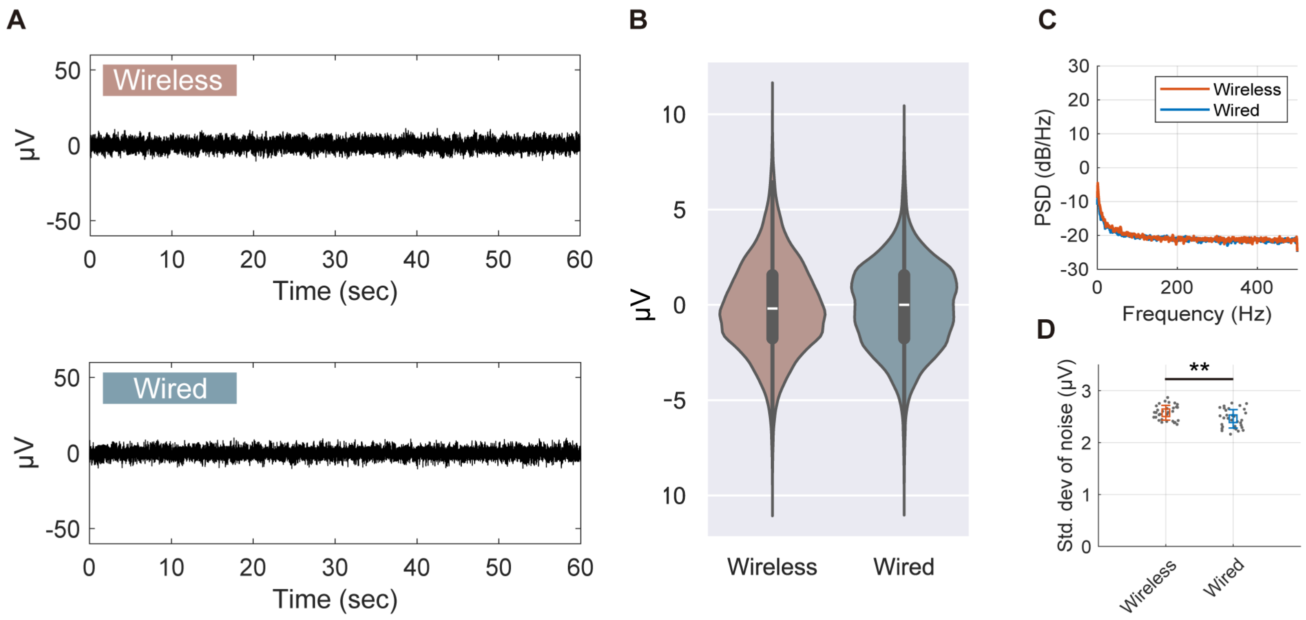

Supplementary Figure 3. Comparison with a wired recording device (benchtop).
A: Example waveforms of baseline (internal) noise for wireless and wired headstages. **B**: Violin plots showing the voltage distribution for baseline noise recorded by the wireless and wired headstages over 60 seconds. **C**. Power spectral densities of the internal noise for both wireless and wired headstages. **D**: Distribution of the standard deviation of internal noise across all 32 channels (n = 32 channels for both headstages). $^{**}p < 0.01$; The p-value was calculated using a two-tailed Welch's t-test.

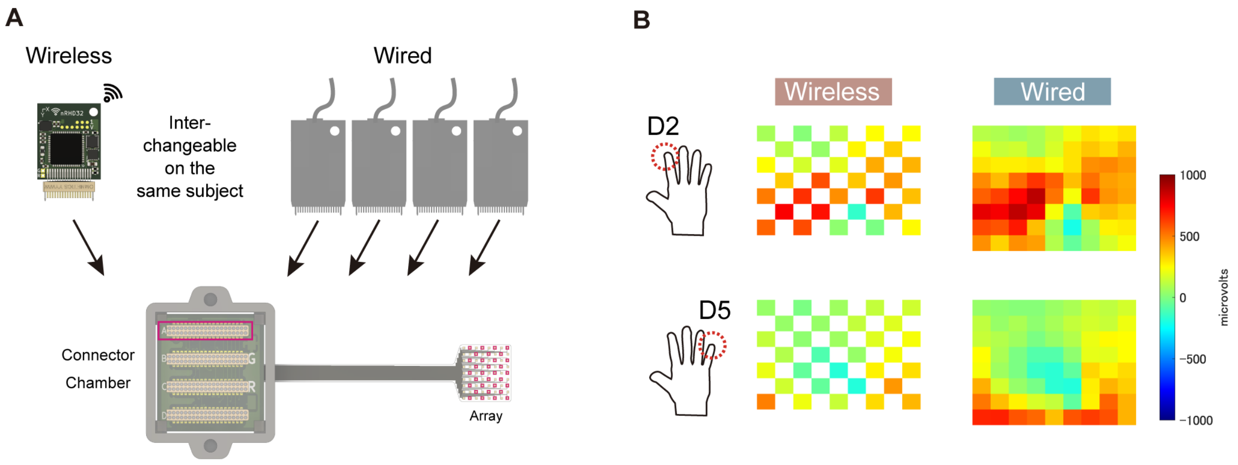

Supplementary Figure 4. Interchangeable recordings from a single animal using wireless and wired headstages.
A: Schematic representation of an electrode array featuring interchangeable recording capabilities. Four parallel connectors are depicted, with those highlighted in pink connected to electrodes marked in pink on the array (totaling 32 channels). **B**: Potential distribution maps of somatosensory evoked potentials recorded by wireless and wired devices. The top row shows the potential distribution following stimulation of the index finger (D2), while the bottom row illustrates the potential distribution after stimulating the little finger (D5) (t = 15 ms post-stimulation). Different colors represent averaged voltage values (n = 50 stimuli).

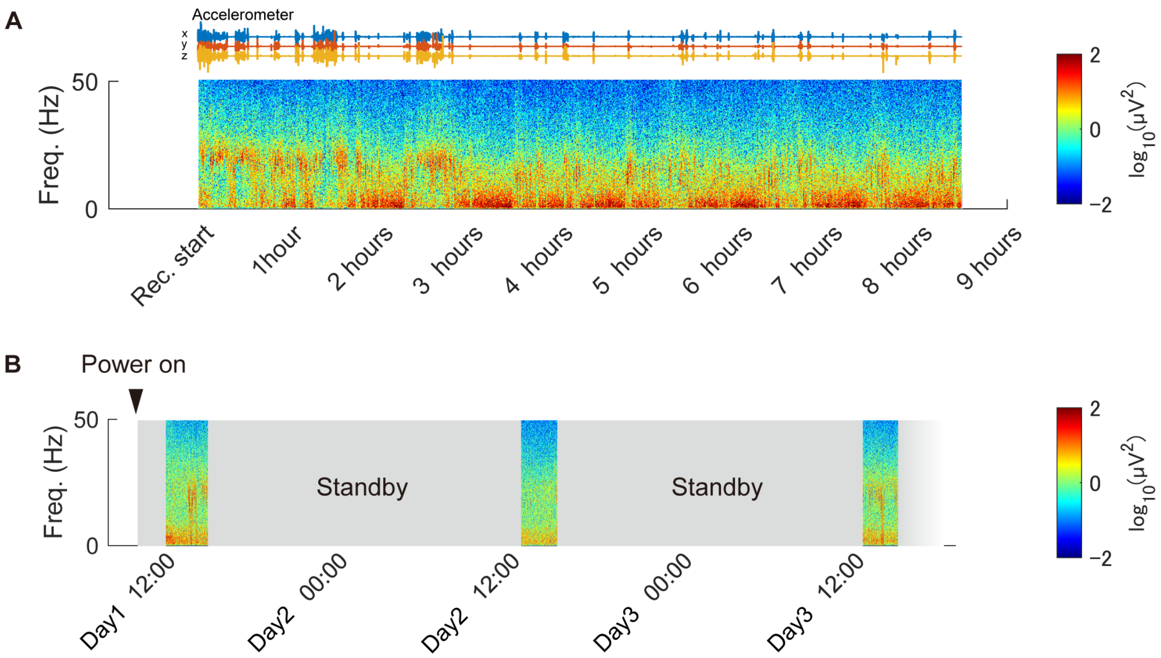

Supplementary Figure 5. Continuous and intermittent in-cage recording using the wireless device (magnified range). This figure presents the same data as shown in Figure 5, with the y-axis magnified to 0–50 Hz to highlight the low-frequency range.

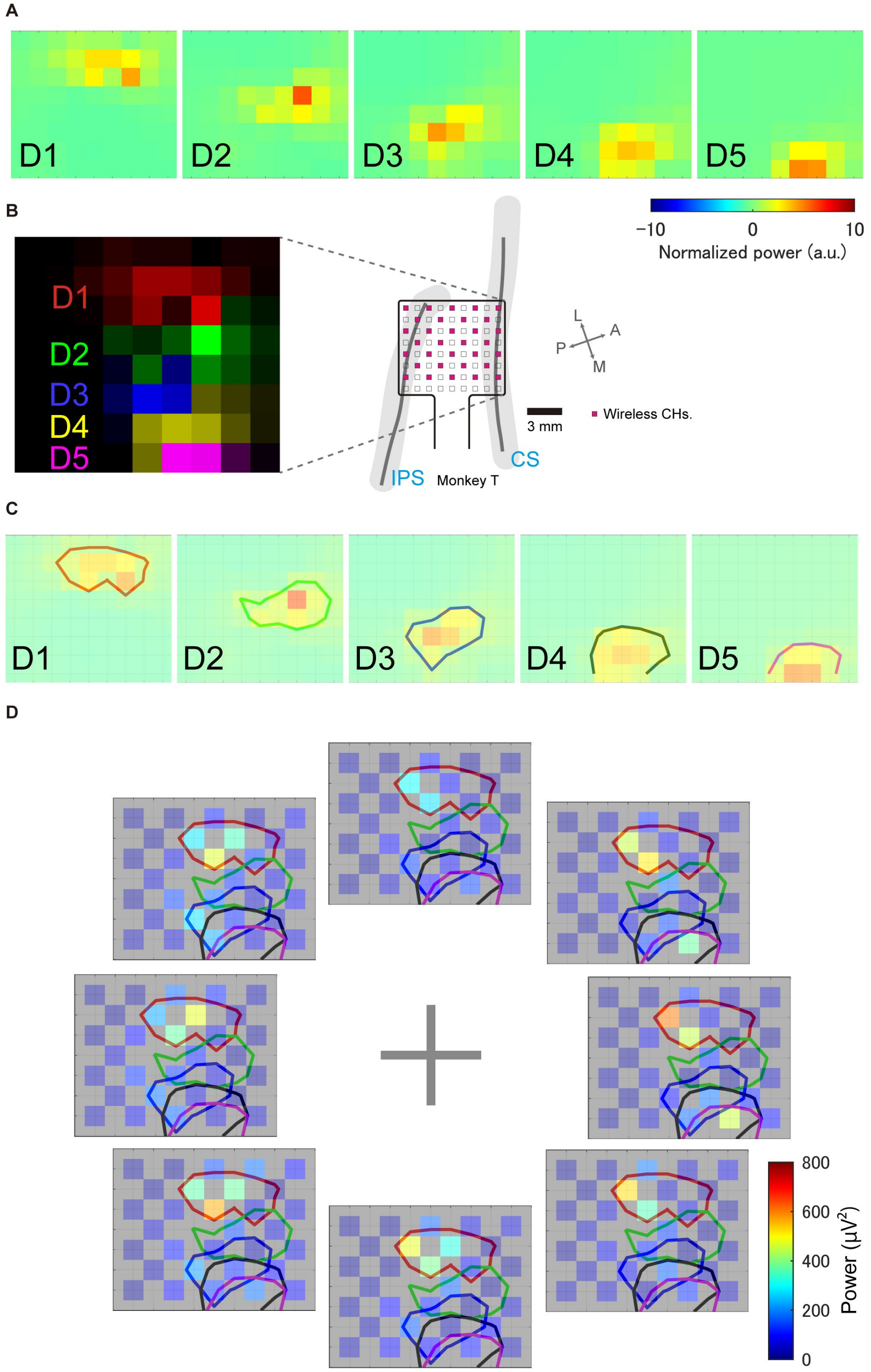

Supplementary Figure 6. Correspondence between visuomotor-task response and somatosensory mapping.
A: Evoked responses observed in the digit representation areas of S1 following electrical stimulation of individual digits (Monkey T, wired recording). Colors represent high-gamma power normalized within the array for each digit. **B**: (Left) Combined high-gamma response map for all digits. (Right) Corresponding electrode layout; pink-filled squares indicate the wirelessly recordable channels. **C**: Contour plots of digit-specific responses. **D**: Response maps for each target direction (from **Figure 6G**), overlaid with contour plots of digit-specific responses.
D1: Thumb; D2: Index; D3: Middle; D4: Ring; D5: Pinky; IPS: Intra-parietal sulcus; CS: Central sulcus.

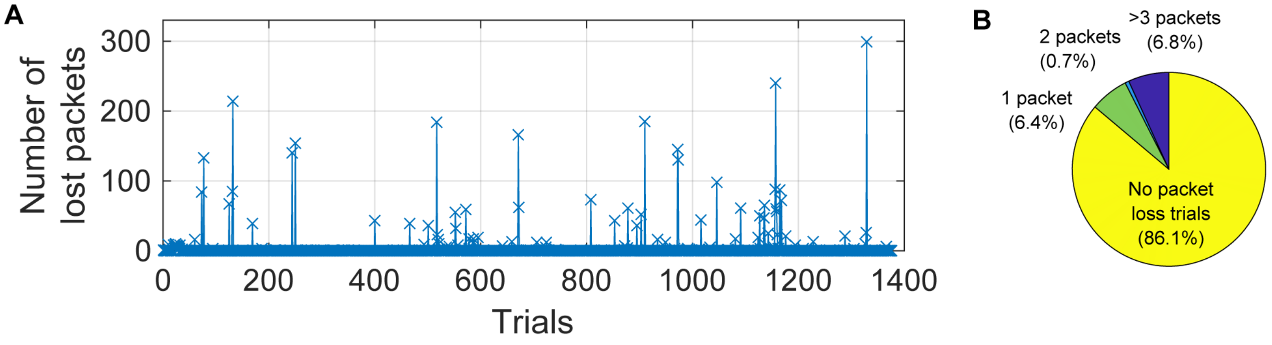

Supplementary Figure 7. Occurrence of packet losses in the in-cage behavioral task.
A: The occurrence of packet losses during the recording session is shown. The vertical axis displays the number of lost packets, whereas the horizontal axis represents the trial numbers in the recording session. Each lost packet corresponds to a loss of 1 ms data. **B**: The proportion of 1,376 trials in the recording session is categorized into trials without any packet loss, trials with only one packet lost, trials with two packets lost, and trials with three or more packets lost.

Supplementary Tables

	**This work**	**Zhou (2019)**[10]	**Romanelli (2018)**[13]	**Fernandez-Leon(2015)**[7]	**Schwarz (2014)**[12]	**Yin (2014)**[8]	**Borton (2013)**[14]	**Miranda (2010)**[31]
**Device type**	Semi-implantable	Semi-implantable	Fully implantable	Semi-implantable	Semi-implantable	Semi-implantable	Fully implantable	Semi-implantable
**Electrode connector**	Omnetics	Omnetics	-	CerePort	Omnetics	CerePort	-	n.s.
**Neural channel count**	32	128[1]	16	96	128[2]	96	100	32
**Maximum sample rate (kHz)**	1	1	0.5	20	31.25	20	20	30
**Size (mm)**	25×16×4[3]	36×33×15	30×29×12[4]	56×42×44	14×22×2[5]	52×44×30	56×42×11	38×38×51
**Device shape**	Upright	Flat	Cubic	Cubic	Flat	Flat	Cubic	Cubic
**Weight (grams)**	1.8[6]	7.4[7]	-	-	-	3.6[7]	44.5[8]	-
**Resolution (bits)**	16	15	-	12	12	-	12	12
**Input referred noise (μVrms)**	2.4[9]	1.6	-	3.93	4.9	2.8	8	3.2
**Battery life (hours) / Battery capacity**	8.5 (100 mAh)	11.3 (500 mAh)	2.6[10] (150 mAh)	n.s. (1600mAh x 2)	>30h (2000 mAh)	>48 (1200 mAh)	7 (n.s.)	33 (1200 mAh×2)
**Power consumption (mW)**	47	172	209	4000	264	61 / 97[11]	90.6	142
**Stimulation**	No	Yes	Yes	No	No	No	No	No

Supplementary Table 1. Wireless devices validated with freely moving primates.

[1] Real-time streaming is limited to 96 channels. [2] 128 channels per single transceiver unit. [3] Excluding housing and battery. [4] Including housing and battery. [5] Size per head stage. Excluding a 43 × 21 × 3.4 transceiver. [6] Excluding housing and battery. [7] Weight of electronics only. [8] Total system weight, including housing and battery. [9] Conforms to the RHD2132 amplifier chip datasheet. [10] Estimated value. [11] Estimated values, 97 mW in high RF mode.

Acronyms and abbreviations (Alphabetical order).

Acronym or Abbreviation	Definition
**ADC**	Analog-to-Digital Converter
**ASIC**	Application-Specific Integrated Circuit
**BCI**	Brain-Computer Interface
**CAD**	Computer-Aided Design
**CMOS**	Complementary Metal-Oxide-Semiconductor
**CPU**	Central Processing Unit
**DC**	Direct Current
**ECoG**	Electrocorticography
**FFT**	Fast Fourier Transform
**FPGA**	Field-Programmable Gate Array
**GPIO**	General Purpose Input/Output
**I/O**	Input/Output
**I2C**	Inter-Integrated Circuit
**IC**	Integrated Circuit
**IMU**	Inertial Measurement Unit
**IR**	Infrared
**LDO**	Low Dropout (voltage regulator)
**LED**	Light-Emitting Diode
**LFP**	Local Field Potential
**Li-Po**	Lithium-Polymer
**mAh**	milliampere hour
**MCU**	Microcontroller Unit
**NHP**	Non-Human Primate
**PC**	Personal Computer
**PCB**	Printed Circuit Board
**PSD**	Power Spectral Density
**RAM**	Random Access Memory
**RF**	Radio Frequency
**RFSoC**	Radio Frequency System-on-Chip
**S1**	The Primary Somatosensory Cortex
**SEP**	Somatosensory Evoked Potential
**SPI**	Serial Peripheral Interface
**USB**	Universal Serial Bus
